# Supplementary material for: High-throughput gene expression analysis with TempO-LINC sensitively resolves complex brain, lung and kidney heterogeneity at single-cell resolution
Source: Sci Rep. 2024 Dec 28;14:31285. doi: 10.1038/s41598-024-82736-6 (PMC11682069; doi:10.1038/s41598-024-82736-6)
Supplement: Supplementary file 1 — Supplementary Material 1 [file 41598_2024_82736_MOESM1_ESM.pdf]

Supplementary Information

High-throughput gene expression analysis with TempO-LINC sensitively resolves complex brain, lung and kidney heterogeneity at single-cell resolution

Dennis J. Eastburn[1#], Kevin S. White[1], Nathan D. Jayne[1], Salvatore Camiolo[1], Gioele Montis[1], Seungeun Ha[1], Kendall G. Watson[1], Joanne M. Yeakley[1], Joel McComb[1], Bruce Seligmann[1]

[1] BioSpyder Technologies, Inc., Carlsbad, CA

[#] To whom correspondence should be addressed: denniseastburn@biospyder.com

Single-cell analysis pipeline tool

The LINC analysis tool is used to demultiplex the reads generated by a LINC sequencing library and align the reads corresponding to each single cell. The main input of the software is a set of paired-end reads (hereafter referred to as read 1 and read 2, where read 1 is 50 nucleotides in length and read 2 is 100 nucleotides in length), a list of barcodes used during the LINC experiment and the probe sequences corresponding to the TempO-Seq panel used for the assay. The tool relies on four steps, which are described below.

Step1: Cell clustering and technical noise suppression

This tool takes input from a paired-end sequencing run, together with a reference probe hash table, the corresponding annotation file in gtf format and a list of barcode sequences used during the LINC library preparation.

During the first step, the sequences are classified into cell clusters. Briefly, every read 2 sequence is searched for the following subsequences that are stored in memory: (a) the UMI specific sequence, the barcode used at the third stage of barcoding, the one used at the second stage of barcoding and the barcode used at the first stage of barcoding. The three barcodes are then joined together (hereafter referred to as the barcode combination) to form the ID of the cells. At this stage up to one mismatch is allowed between the found barcodes and the known list of TempO-LINC barcode sequences. This mismatch allowance allows a small amount of technical noise to be corrected for with all 48 barcodes (minimum hamming distance of 4).

All the barcode combinations, together with their number of occurrences in read 2, are reported into an output file called barcode_combination.csv. This file is sorted by the total number of assigned reads and the inflection point is detected. Such inflection point is used to identify the number of reads that differentiates real cells from signal noise. Once this threshold has been identified, only the barcode combinations with a number of occurrences exceeding the threshold are retained for the downstream analyses.

Step 2: Read alignment

The reads in the read 1 file that correspond to each cluster identified in the previous step are extracted and aligned against the probe sequences corresponding to the panel used during the LINC experiment. After the alignment is complete a count table is generated using a feature counter.

Step 3: Transcript alignment

The reads that are extracted in the previous step are collapsed into the original transcripts they were generated from, by exploiting the UMI sequences retrieved during step 1. A supersequence composed of the UMI, the barcode combination and the corresponding read 1 is generated for all the reads used in Step 2. All the perfect replicates (no mismatch in the pairwise comparison, e.g. hamming distance = 0) are removed leaving just one copy (the tool fastuniq is used at this stage). The remaining sequences are collapsed to clusters featuring a sequence homology of 97% (this is performed by using the tool cd-hit). Again, for each cluster, only one sequence is randomly chosen and all the others are considered PCR clones. The collapsed reads are then again aligned against the probe sequences similarly to what is done with the original reads in Step 2.

Step 4: Generation of the output files

All the read count tables obtained in Step 2 are merged together in the final probe read count table. The same process is performed for all the transcript count tables obtained in Step 3 thus generating the UMI probe count table. The counts of probes targeting the same gene are summed up to form the gene read and umi count tables. At this stage all the QC metrics are calculated and reported in the sequencing_stats.csv file.

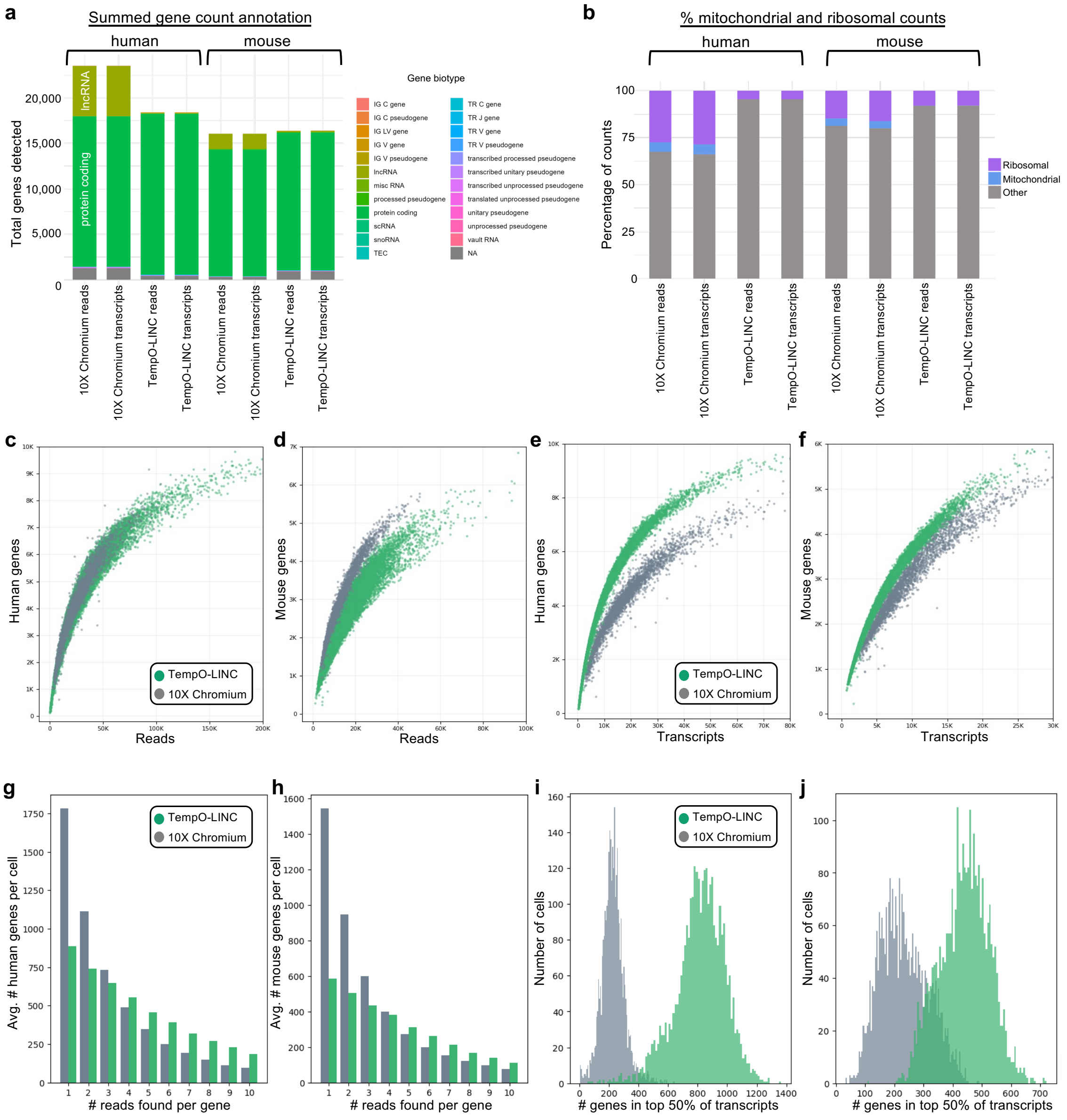

Supplemental Figure 1. Platform comparison between TempO-LINC and 10X Genomics Chromium single-cell gene expression assays. Publicly available HEK293 and mouse NIH3T3 single-cell gene expression datasets generated using the 10X Genomics Chromium (v2) assay (Ding, J. et al., Nat. Biotechnol., 2020) were compared against TempO-LINC data on the same cell types. Replicate experimental datasets from either 10X Chromium or TempO-LINC platforms were combined into a single dataset for the analyses presented in this figure. (a) Summed human and mouse gene counts for all single-cell data from either 10X Chromium or TempO-LINC platforms are displayed in the bar plot. The genes were annotated based on their Ensembl gene biotype designation and showed that although TempO-LINC identifies more protein coding genes, Chromium data has a greater number of detected lncRNAs that are not targeted in the TempO-LINC DO pool. (b) Percentages of ribosomal and mitochondrial reads or transcripts are provided for both platforms. For the 10X Chromium dataset, up to 33% of human transcript counts map to ribosomal or mitochondrial genes, whereas that number is <4% for TempO-LINC. Figure panels c-j show TempO-LINC data colored green and 10X Chromium data is shown in gray. Single-cells were plotted showing the number of genes detected vs the number of human and mouse reads (c,d) or transcripts (e,f) per cell. (g,h) Following selection of cells at the same total read depth, the average number of genes per cell are plotted relative to the number of reads found for that gene. Equal numbers of cells found in bins centered on 50,000 reads/ human cell or 35,000 reads/mouse cell from either Chromium or

TempO-LINC platforms were used for this comparison. The data demonstrate that TempO-LINC cells have fewer genes that are “weakly” detected by only one or two reads. (i,j) To examine the effects of attenuation and low ribosomal and mitochondrial transcript counts associated with TempO-LINC data, we examined the single-cell P50 values. The P50 metric is defined as the number of highly expressed genes within a single-cell capturing the top 50% of transcript counts. For this analysis, equal numbers of cells were examined from TempO-LINC and Chromium data (3,283 cells for human and 2,945 cells for mouse). In both human (i) and mouse (j) cells, the TempO-LINC number of P50 genes is much higher than those from 10X Chromium profiled cells. This indicates that TempO-LINC transcript counts are more broadly and evenly distributed across a larger number of genes. For Chromium data, a smaller number of highly expressed genes take up the majority of the transcript counts resulting in lower gene discovery rates for a given number of transcripts.

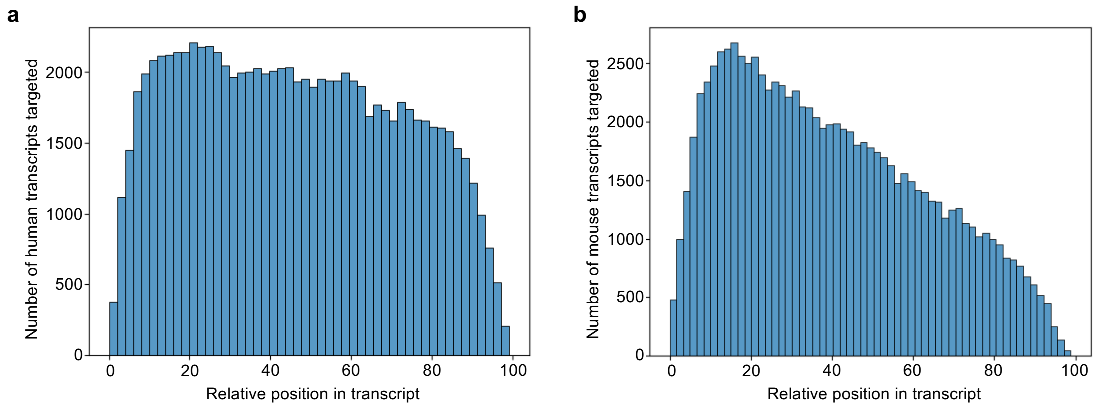

Supplemental Figure 2. TempO-LINC probe positional analysis on mRNA. Bar plots showing the relative targeting position of DO probes within their target mRNAs with “0” representing the 5’ end and “100” the 3’ end of the message. (a) Analysis of the Human Whole Transcriptome v2.1 (22,533 probe pairs, 19,683 genes) DO pool. (b) Distribution of the Mouse Whole Transcriptome v1.1 (30,146 probe pairs, 21,400 genes) DO pool.

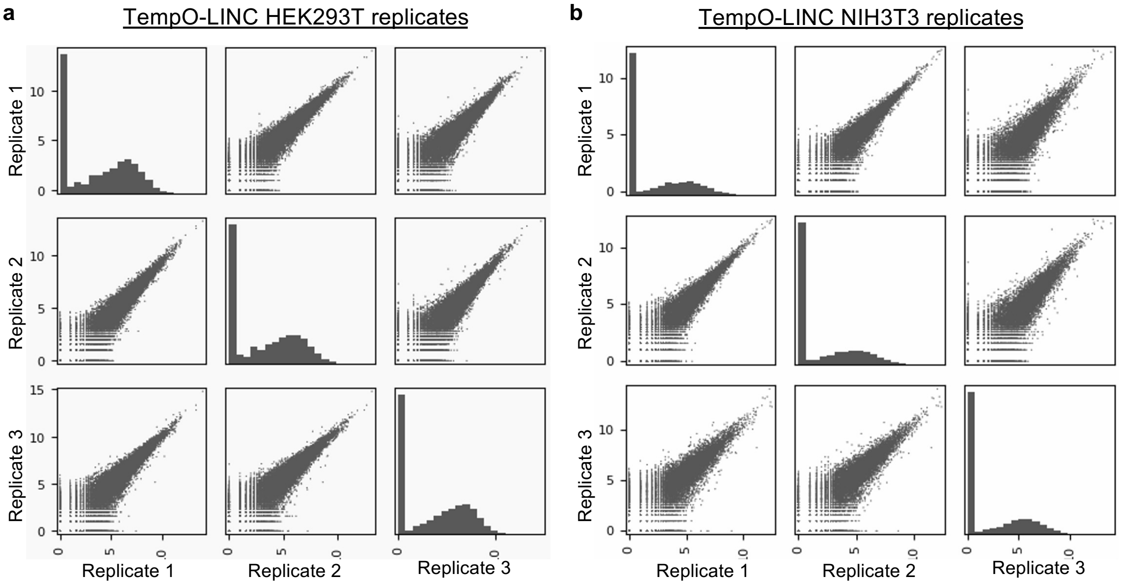

	Replicate1	Replicate2	Replicate3
Replicate1	1.000	0.957	0.946
Replicate2	0.957	1.000	0.946
Replicate3	0.946	0.946	1.000

	Replicate1	Replicate2	Replicate3
Replicate1	1.000	0.947	0.924
Replicate2	0.947	1.000	0.932
Replicate3	0.924	0.932	1.000

Supplemental Figure 3. TempO-LINC reproducibility. (a) The scatterplot matrix shows the correlation between the sum of the log2 transformed read counts from 50 random human HEK293T cells across three TempO-LINC replicates. Plots reported in the diagonal of the matrix show the distribution of the log2 transformed read counts for each replicate (x axis = log2 transformed sum of counts from 50 random cells, y axis: number of genes). Pearson *r* values are reported in the table below the scatterplot matrix. (b) Identical scatterplot matrix shown for the sum of log2 transformed read counts from 50 random mouse NIH3T3 cells in the three replicates.

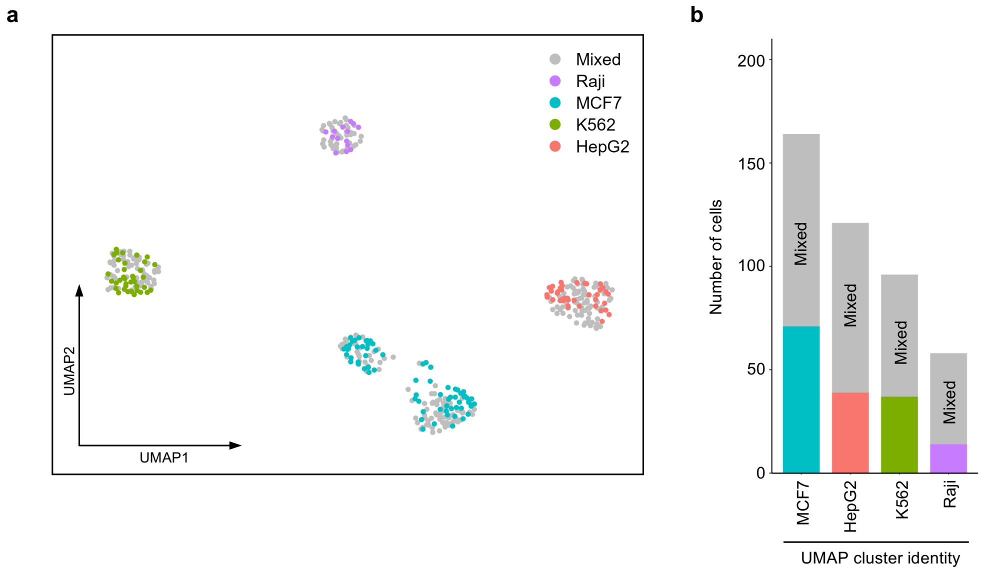

Supplemental Figure 4. Mixed human cell line analysis. (a) Human MCF7, Raji, K562 and HepG2 cell lines as well as a mixture of all four cell lines (Mixed) were simultaneously barcoded with TempO-LINC for single-cell transcriptome profiling. 439 total cells were identified with a UMAP plot showing four distinct populations of cells identified following unsupervised clustering. Based on the Barcode 1 identity, cells for mixed and independent cell line samples were color coded on the UMAP, demonstrating that TempO-LINC can correctly resolve cell type identity from mixed populations of human cells. (b) Bar plot showing the number of cells associated with each of the 4 major UMAP populations. The number of cells associated with "Mixed" (gray fraction) or cell line-specific barcode sample identities can be seen for each bar on the plot. The precise cell numbers were: 278 total Mixed cells identified and found distributed into all 4 of the cell cluster populations, 71 MCF7 cells, 39 HepG2 cells, 14 Raji cells and 37 K562 cells.

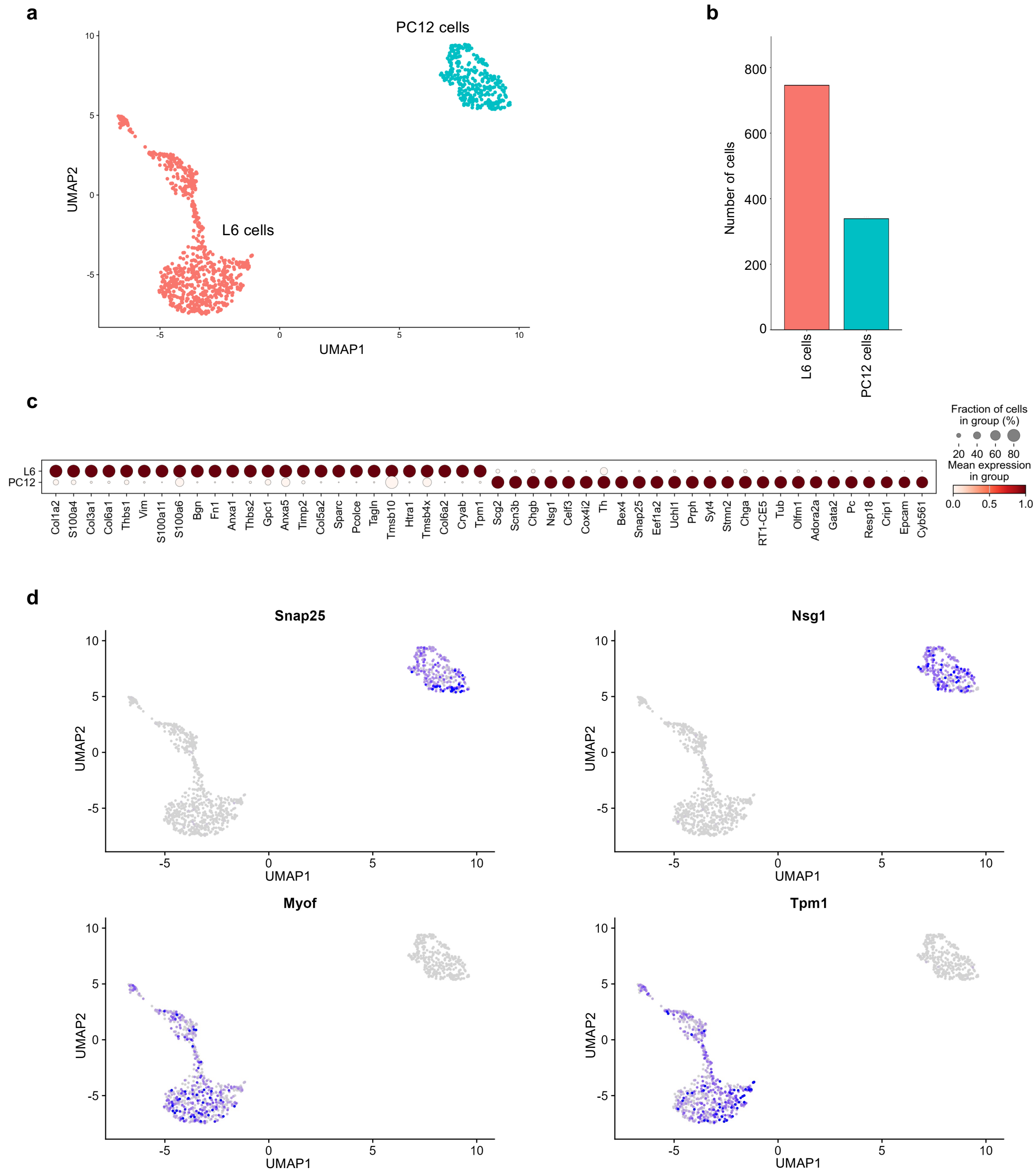

Supplemental Figure 5. Single-cell transcriptional profiling of rat cells. (a) Rat L6 myoblasts and PC12 neuronal cells were simultaneously barcoded with TempO-LINC. 1,085 total cells were identified following demultiplexing and cell filtering. The UMAP plot shows two distinct populations of cells identified following unsupervised clustering in Seurat. (b) Bar plot of the number of cells associated with each of the UMAP populations. (c) Dot plot showing the cluster specific expression patterns of the top 25 most significantly upregulated genes (ranked by p-value) for both L6 and PC12 clusters. (d) Feature plots of 4 representative biomarkers demonstrate expression levels within clustered single cells.

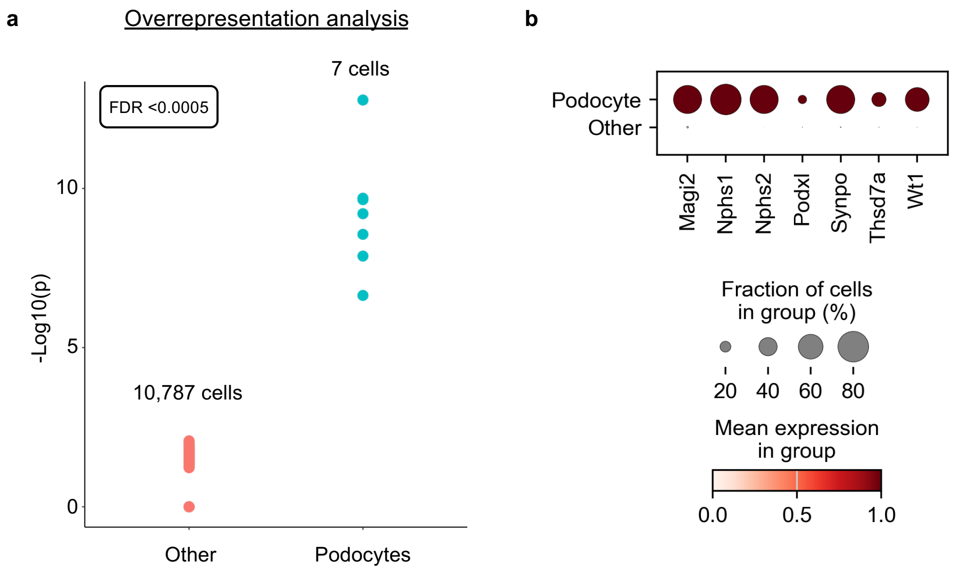

Supplemental Figure 6. Identification of podocytes from kidney tissue. (a) Identification of podocytes within the mouse kidney was performed using overrepresentation analysis utilizing a seven podocyte marker gene set consisting of Nphs1, Nphs2, Podxl, Wt1, Magi2, Synpo and Thsd7a. The false discovery rate (FDR) value was calculated using the Benjamini & Hochberg adjustment method. (b) Dot plot analysis showing expression of seven known molecular markers for podocytes across either the identified podocytes or the 10,787 "other" cells resulting from the overrepresentation analysis in panel "a".

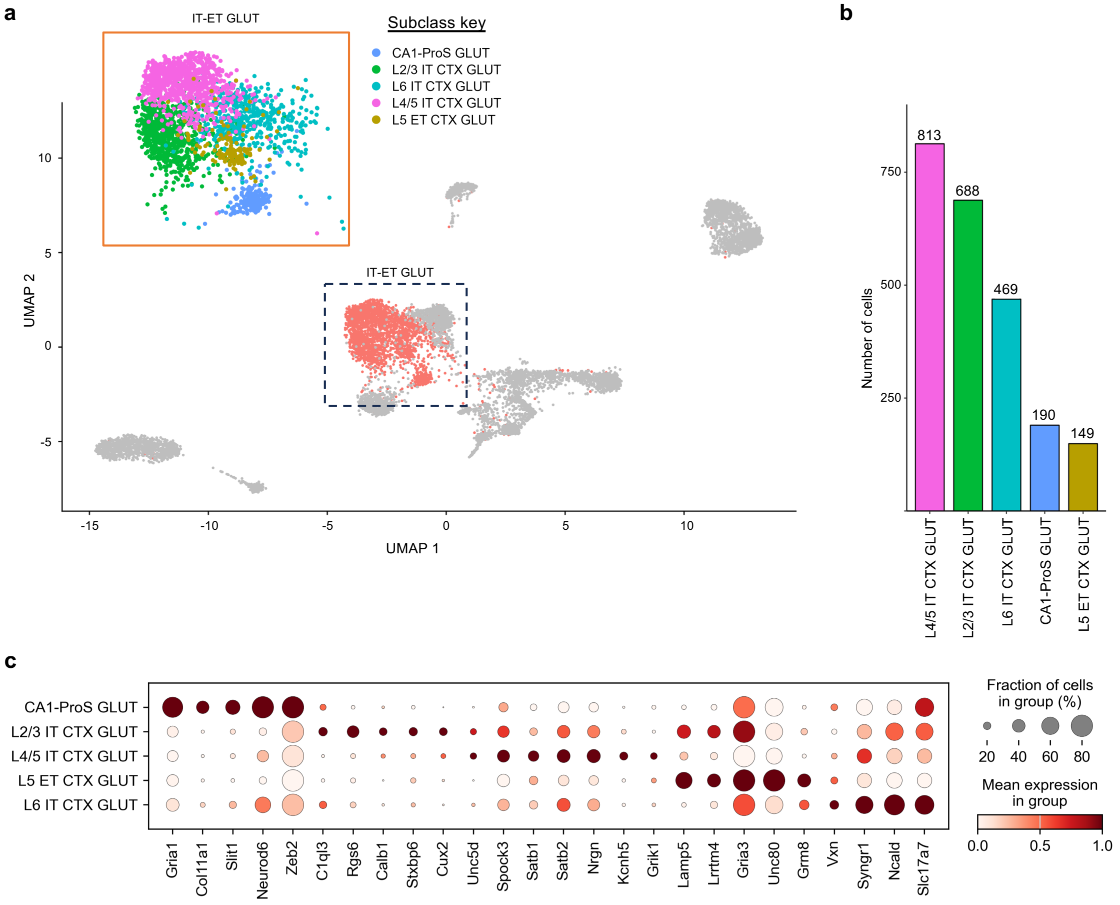

Supplemental Figure 7. Neuronal subclass identification within the IT-ET GLUT cluster. (a) Increasing the cluster resolution on the Seurat generated Figure 4 UMAP demonstrates that 5 additional neuronal cell subclusters can be identified within the IT-ET GLUT population. Dashed box indicates the region on the UMAP in Figure 4 that is magnified in the orange box. IT-ET subclasses identified and annotated include: L2/3 IT CTX GLUT, L4/5 IT CTX GLUT, L6 IT CTX GLUT, L5 ET CTX GLUT and CA1-ProS GLUT. (b) Cell numbers for each subclass are plotted. (c) Dot plot showing expression of 26 subclass-specific biomarkers within the 5 identified IT-ET GLUT subclusters.
